# CD248-expressing cancer-associated fibroblasts induce epithelial–mesenchymal transition of non-small cell lung cancer via inducing M2-polarized macrophages

**DOI:** 10.1038/s41598-024-65435-0

**Published:** 2024-06-21

**Authors:** Jing Xiao, Zeyang Yang, Siyu Wang, Xinlei Liu, Yun Wang, Zuquan Hu, Zhu Zeng, Jieheng Wu

**Affiliations:** 1 Department of Immunology, Guizhou Medical University, Siya Road, Guiyang, 561113 China; 2 College of Stomatology, Guizhou Medical University, Guiyang, 561113 China; 3 Guizhou Prenatal Diagnosis Center, The Affiliated Hospital of Guizhou Medical University, Guiyang, 550001 China; 4 Immune Cells and Antibody Engineering Research Center of Guizhou Province, Key Laboratory of Biology and Medical Engineering, Guizhou Medical University, Guiyang, 561113 China; 5 Key Laboratory of Infectious Immune and Antibody Engineering of Guizhou Province, Engineering Research Center of Cellular Immunotherapy of Guizhou Province, School of Biology and Engineering/School of Basic Medical Sciences, Guizhou Medical University, Guiyang, 561113 China; 6 The State Key Laboratory of Cancer Biology, Department of Biochemistry and Molecular Biology, The Fourth Military Medical University, Xi’an, 710032 China; 7 Tumor Immunotherapy Technology Engineering Research Center of Guizhou Medical University, Guizhou Medical University, Guiyang, 561113 China

**Keywords:** CD248, Cancer-associated fibroblasts, Non-small cell lung cancer, M2 macrophages, EMT, Cancer microenvironment, Non-small-cell lung cancer

## Abstract

Non-small cell lung cancer (NSCLC)-originating cancer-associated fibroblasts (CAFs) expressing CD248 regulate interaction with immune cells to accelerate cancer progression. Epithelial–mesenchymal transition (EMT) is a key feature of metastatic cells. In our pervious study, we found that CD248^+^CAFs activated M2-polarized macrophages, enhancing the progression of NSCLC. However, it is yet unclear how CD248^+^CAFs inducing M2-polarized macrophages induce EMT program in NSCLC cells. Herein, we examined CD248 expression from CAFs derived from NSCLC patient tumour tissues. Furthermore, we determined the influence of CD248 knock down CAFs on macrophages polarization. Next, we explored the influences of CD248-harboring CAFs-mediated M2 macrophage polarization to promote NSCLC cells EMT in vitro. We constructed fibroblasts specific CD248 gene knock out mice to examine the significance of CD248-harboring CAFs-induced M2-polarized macrophages to promote NSCLC cells EMT in vivo*.* Based on our analysis, CD248 is ubiquitously expressed within NSCLC-originating CAFs. CD248^+^CAFs mediated macrophages polarized to M2 type macrophages. CD248^+^CAFs induced M2 macrophage polarization to enhance NSCLC cells EMT both in vivo and in vitro. Our findings indicate that CD248-harboring CAFs promote NSCLC cells EMT by regulating M2-polarized macrophages.

## Introduction

Lung cancer (LC) is highly prevalent among all cancers. Its prevalence and mortality rates rank second and first, respectively, among all malignant tumors. NSCLC constitutes ~ 80–85% of newly diagnosed LC incidences^1^. About 75% of NSCLC patients exhibit evidence of regional or distal metastasis, and only 15% of metastatic NSCLC patients live for at least 5 years post diagnosis^2,3^. Exploring the molecular mechanisms underlying NSCLC progression remains the focus of cancer research.

Cancer formation is multi-tiered and involves the migration of cancerous cells to distant locations. Among its essential regulations is the EMT process, which enables cancerous cells to break free of their surrounding and translocate to other regions of the body. Under metastatic conditions, epithelial cells lack E-cadherin expression, and have vastly diminished cell–cell adhesion and apico-basal polarization, with markedly enhanced vimentin content and cellular motility^4–6^. Therefore, the conversion of E- to N-cadherin is a crucial stage in metastasis^7,8^. Nonetheless, the underlying mechanisms that regulate this complicated EMT network in NSCLCs remain completely unelucidated. Emerging reports suggest that the tumor microenvironment (TME) is a strong modulator of EMT in tumor cells^9^. As an essential TME component, CAFs can interact with other stromal or tumor cells and modulate immune evasion, drug resistance, and tumor angiogenesis^10,11^. Due to the wide range of fibroblasts, CAFs have an elevated degree of phenotypic heterogeneity, and the functions of CAFs with varying phenotypes are also different. Therefore, it is necessary to find relatively specific markers on CAFs, which are crucial for the study of CAFs in NSCLC cells EMT process.

CD248 (endosialin/tumor endothelial marker 1) is a type I transmembranal glycoprotein. The majority of tumor neovascular endothelial cells express CD248 and not normal vascular endothelial cells. CD248 is also strongly expressed in diverse sarcomas, neuroblastomas, skin cancers, breast cancers, and additional tumors^12^. In addition, activated fibroblasts express CD248, which is involved in controlling fibroblast proliferation and migration^13^. In our previous study, we demonstrated that CD248^+^CAFs secreting CXCL12 mediated macrophages polarized to M2 type macrophages promotes NSCLC progression^14^. However, the role by which CD248^+^CAFs inducing M2-polarized macrophages to induce EMT program in NSCLC cells remains unclear.

Herein, we demonstrated that CD248 is ubiquitous within NSCLC-originating CAFs. CD248^+^CAFs mediated macrophages polarized to M2 type macrophages. CD248^+^CAFs activated M2-polarized macrophages to accelerate NSCLC cells EMT both in cellular and animal models. Our study emphasizes the importance and possible signaling networks of CD248^+^CAF in NSCLC progression and offers a novel strategy for improving the survival time of NSCLC patients.

## Materials and methods

### Study approval

NSCLC and matched healthy specimens collection was approved by the Clinical Research Ethics Committee of Guizhou Medical University and conducted in accordance with relevant ethical regulations. Informed consent was obtained from each patient, and the study protocol (no. 2023LL-23) was approved by the Clinical Research Ethics Committee of Guizhou Medical University and complied with all relevant ethical guidelines. Animal procedures were performed in accordance with protocols approved by the Institutional Animal Care and Use Committee (IACUC) of Guizhou Medical University (no. 2300105). All experiments were performed according to the relevant guidelines, regulations, and ARRIVE guidelines.

### Tumor tissues

Tissue samples, both NSCLC and matched healthy specimens, were obtained from The Affiliated Hospital of Guizhou Medical University. Guizhou Medical University granted ethical permission for this study (approval number 2023LL-23), and all subjects provided signed documentation of their informed consent.

### Mice

Suzhou Cyagen Co., Ltd., produced mice with floxed cd248 or fsp-1-Cre. *cd248*^*fl/fl*^*fsp-1*^+*/*+^ (WT) and *cd248*^*fl/fl*^*fsp-1*^*cre/*+^ (cKO) mice were established via crossing floxed cd248 mice with fsp-1-Cre mice. All animals were bred and housed in pathogen-free environments, with free access to standard laboratory diet, at 12 h light/dark cycle, 22 ± 1 °C, and 55% ± 5% relative humidity. All rodents were housed in individual cages, had a C57BL/6 genetic background, and were aged between 6–12 weeks during experimentation. Cre-negative littermate mice served as controls. All animal protocols received ethical approval from the Guizhou Medical University.

### Cell lines and co-culture assay

Human cell lines, NSCLC A549 and NCI-H460 and monocyte THP-1, and murine LC cell line, Lewis cell (LLC) cell were obtained from the ATCC (Manassas, VA, USA) and verified to be free of Mycoplasma by short tandem repeat (STR) profiling. Cells were grown in Roswell Park Memorial Institute (RPMI) 1640 medium or Dulbecco's Modified Eagle Medium/Nutrient Mixture F-12 (DMEM/F12) (Gibco, Waltham, MA, USA) containing 10% FBS and 1% penicillin–streptomycin (both Invitrogen, Waltham, MA, USA) in a humid incubator at 37 °C with 5% CO_2_. Fibroblasts were isolated and cultivated as previously described^14,15^. We generated CAFs-sh-CD248 cells with sustained CD248 deficiency and CAFs-CD248OE cells with sustained CD248 overexpression by lentivirus transduction.

To conduct polarization evaluation, THP-1 monocytes were 24 h treated with phorbol 12-myristate 13-acetate (PMA, 100 ng/mL) to generate M0 macrophages.

To perform coculture assessment, we employed conditioned medium (CM). CAFs-sh-CD248 or CAFs-sh-CON were grown together with THP-1 cells in 6-well plates for 48 h. The corresponding supernatants were introduced to A549 and NCI-H460 cells over a 48 h period prior to migratory evaluation via Transwell assay.

### Western blot (WB)

We employed primary antibodies as follows for our WB analysis: anti-human CD248 (CST, #47948, Louisville, KY, USA), anti-FAP (Servicebio, #GB11096), anti-α-smooth muscle actin (SMA) (Servicebio, #GB11044), anti-PDGFRβ (Thermo Fisher, #MA5-15143), anti-Smad2/3 (Servicebio, #GB111844), anti-p-Smad2/3 (CST, #8828), anti-TWIST (Absin, #abs131127), anti-vimentin (Servicebio, #11192), anti-N-cadherin (Absin, #abs131133), anti-E-cadherin (Absin, #abs130068), and anti-β-actin (Servicebio, #11002). We separated proteins using sodium dodecyl sulfate–polyacrylamide gel electrophoresis (SDS-PAGE)-based separation, prior to transfer to polyvinylidene difluoride membranes, then, membranes were blocked with Tris-buffered saline/Tween (TBST) containing 5% bovine serum albumin for 2 h at room temperature (RT), before exposure to secondary antibodies conjugated with horseradish peroxidase (Servicebio, #GB23303; #GB23301). We conducted three separate experimentations, and analyzed the results. Protein bands were visualized with multi-image light cabinet filter positions. To conserve both antibodies and membrane materials, the blots were cut prior to hybridization with antibodies. Original images of blots are provided in Supplementary Figs. 1–3.

### Real-time quantitative polymerase chain reaction (RT-qPCR)

Total RNA was isolated with a MiniBEST Universal RNA Isolation Kit (Takara, #9767, San Jose, CA, USA), prior to conversion to cDNA with PrimeScript RT Master Mix (Takara, #RR063A), and qPCR was conducted via a TB Green Premix Ex Taq II Kit (Takara, #RR820). Supplementary Table details the overall procedure.

### Immunofluorescence (IF) staining

Antibodies used for the IF-based evaluation included anti-human CD248 (CST, #47948), anti-α-SMA (Abcam, #ab5694), anti-CD206 (CST, #24595), and anti-CD68 (CST, #97778). We employed Tyramide Signal Amplification (TSA) technology for evaluation of CD248, α-SMA, CD206, CD68, E-cadherin and N-cadherin expression and colocalization in tumor tissue slices during multiple immunofluorescence (IF) labeling. Slides were incubated in 5% BSA at RT for 2 h, then for 15 min in 3% H_2_O_2_. Step 1: A 1 h overnight (ON) exposure to primary antibodies at 4 °C. Step 2 involved a 50-min incubation in HRP-conjugated secondary antibody at RT following washing in TBST. In Step 3, slides received a 10 min exposure to TSA fluorophores at RT. In step 4, EDTA antigen retrieval buffer was boiled to remove the antibody-TSA complex. Steps 1–4 were duplicated until all the antibodies stained the corresponding antigens. Laser scanning confocal microscopy was used to counterstain nuclei with DAPI.

Immunofluorescent cells were fixed for 15-min in 4% paraformaldehyde (PFA) at RT, then permeabilized for 10-min in 0.2% Triton X-100 prepared in PBS, followed by incubation with 5% BSA for 2 h at RT. The cells were then probed with anti-CD248 (1:300) and anti-αSMA (1:500) ON at 4 °C, followed by a 1 h exposure to secondary antibodies conjugated with Alexa-Fluor (Servicebio). PI counterstained the nuclei, and the cells were examined and imaged using a laser-scanning confocal microscope.

### ELISA

Place CAFs-sh-CON/CAFs-sh-CD248 cells in the bottom Transwell chamber, and place THP-1 cells in the upper compartment were incubated for 48 h. After 48 h, THP-1 cells were grown in fresh serum-free media, followed by THP-1 cell supernatant harvest 24 h later for assessment using TGF-β ELISA kits (R&D Systems, # DB100C, USA), as directed in kit protocols.

### Mouse tumor experiments

The Guizhou Medical University approved this animal study. To mimic lung metastasis, 5 × 10^5^ LLC cells were intravenously administered into age- and sex-paired WT and cKO mice in 200 µL of PBS. Tumor volume was computed as follows: V (mm^3^) = a × b^2^/2, where a and b represent long and short diameters, respectively. At the end of experiment, mice underwent euthanasia and their tumors were photographed. Paraffin sections were prepared for H&E and IF staining of tumor tissues. All efforts were made to minimize animal suffering and to reduce the number of animals used. Mice were anesthetized with isoflurane (1–5%) for induction and maintenance of anesthesia for 24 h after the last stimulation. The mice were subsequently sacrificed using CO_2_ and the material was collected.

### Statistical analysis

Data analyses utilized Excel 2016. Normality and equality tests of variance were performed on the quantitative data before the analysis. *p-*values < 0.05 were considered statistically significant.

### Ethics approval and consent to participate

Animal studies were conducted in accordance with the Animal Use Protocol approved by Guizhou Medical University (approval no. 2300135). The Affiliated Hospital of Guizhou Medical University provides biopsies of human NSCLC and matched healthy tissues for comparison. All participants provided written informed consent before the research began, and the study was authorized by the ethics committee of the same institution (approval no. 2023LL-12).

## Results

### Ubiquity of CD248 in NSCLC-originating CAFs.

**Figure 1:**
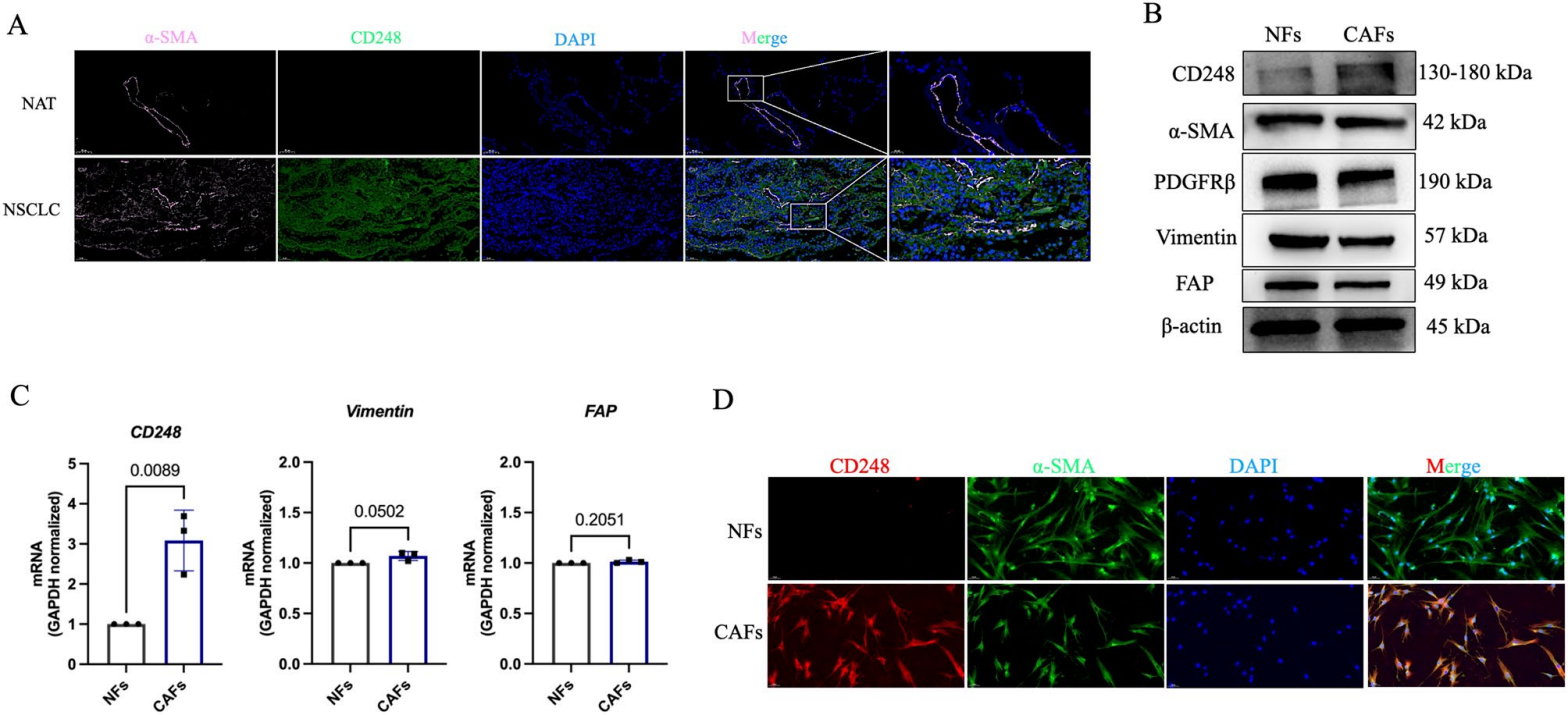
CAFs from NSCLC strongly express CD248. (**A**) Typical representation of dual immunofluorescence (IF) staining demonstrating CD248 and α-SMA colocalization in human NSCLC and normal adjoining tissues (NATs). Scale bar, 50 μm, 20 μm. (**B**) CD248, α-SMA, PDGFRβ, Vimentin and FAP in extracted CAFs and NFs, as determined by western blotting. (**C**) qPCR analysis demonstrating the *CD248*, *FAP* and *Vimentin* contents in CAFs and NFs. We conducted three separate experiments, and the results were analyzed. Data provided as mean ± SD. (**D**) IF staining demonstrating CD248 and α-SMA expression in CAFs and NFs. Scale bar, 50 μm. To conserve both antibodies and membrane materials, the blots were cut prior to hybridization with antibodies. Original images of blots are presented in Supplementary Fig. 1.

To determine the CD248 profile in NSCLC, immunofluorescence (IF) assessments were performed on NSCLC and normal tissues. Positive expression of CD248 was observed in NSCLC tissues (Fig. 1A), however, only minor expression was observed in non-neoplastic tissues (Fig. 1A). Next, we extracted fibroblasts from tumour tissues (referred to as CAFs) and normal adjacent tissues (referred to as NFs) derived from NSCLC patients. WB and RT-qPCR were utilized to validate that extracted CAFs and NFs expressed FAP, Vimentin, and -SMA; however, CAFs expressed CD248 at a significantly higher level than NFs (*p* = 0.0089) (Fig. 1B,C). The results of IF staining demonstrated that the fluorescence was quite visible among CAFs, but not NFs cells (Fig. 1D). Based on these findings, CD248 is ubiquitous in CAFs derived from NSCLC tissues.

### CD248-harboring CAFs activated macrophage differentiation to generate M2-polarized macrophages

**Figure 2:**
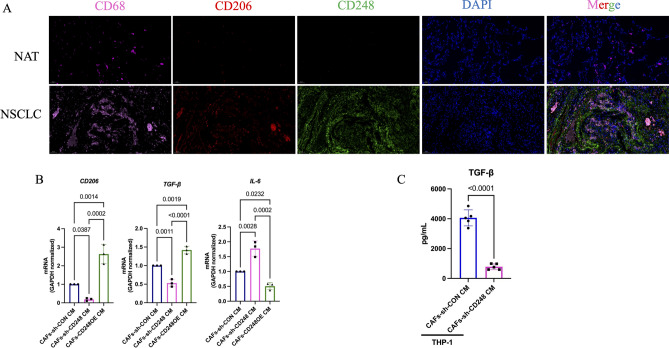
CD248-expressing CAFs induced macrophages polarized to M2-type macrophages. (**A**) Dual immunofluorescence (IF) staining demonstrating CD248, CD68 and CD206 colocalization in human NSCLC and normal adjoining tissues (NATs). Scale bar, 50 μm. (**B**) qPCR analysis showing the levels of *IL-6*, *CD206* and *TGF-β* in M0 macrophages after they were cultured with the CM of CD248 knockdown or control CAFs. Three independent experiments were performed and analysed. Data are provided as the means ± SEMs.* p* values were determined by a two-tailed *t*-test. (**C**) ELISA showing the levels of THP-1 secreting TGF-β in the CM of CD248 knockdown or control CAFs treated. Five independent experiments were performed and analysed. Data are provided as the means ± SEMs. *p* values were determined by a two-tailed *t*-test. *p* < 0.0001.

Macrophages, including inflammatory (M1) and anti-inflammatory (M2) macrophages, exert a great impact on the regulation of tumour progression^14,16^. M1-polarized macrophages (CD68^+^CD206^−^CD163^−^) secrete mainly IL-6, IFN-γ and GM-CSF to exert proinflammatory effects, while M2-polarized macrophages (CD68^+^CD206^+^CD163^+^) secrete mainly IL-10, and TGF-β exerts an anti-inflammatory effect^14,17^. To assess M2-polarized macrophages invasion in NSCLC, tumor and normal tissues were stained using IF. We revealed that the NSCLC/nonneoplastic tissues displayed a noticeable quantity of cells that expressed CD206, specifically M2-polarized macrophages biomarkers. Interestingly, CD248 was absent, and the M2-polarized macrophages invaded the normal tissues (Fig. 2A).

We next assessed the potential CD248-mediated regulation of M2-polarized macrophages using CM from CD248-deficient or control CAFs for treating THP-1 cells. Our analysis revealed that the macrophages co-cultured with CAFs-sh-CD248 CM exhibited reduced levels of the M2 markers *IL-10*, *CD163*, and *CD206* and enhanced *IL-6* (M1 biomarkers), compared to the macrophages maintained in CAFs CM, while the macrophages co-cultured with CAFs-CD248OE CM exhibited higher levels of *IL-10*, *CD163*, and CD206 and a lower level of *IL-6* (Fig. 2B). Next, we used ELISA to detect the secreting TGF-β by M2-polarized macrophages. The results showed that CD248^+^CAFs activated M2-polarized macrophages secreted TGF-β (*p* < 0.0001) (Fig. 2C). These evidences demonstrate that CD248^+^CAFs activated macrophage differentiation to form M2-polarized macrophages.

### CD248-harboring CAFs activated M2-polarized macrophages accelerated NSCLC invasive and migratory activities by inducing EMT program of NSCLC cells

**Figure 3:**
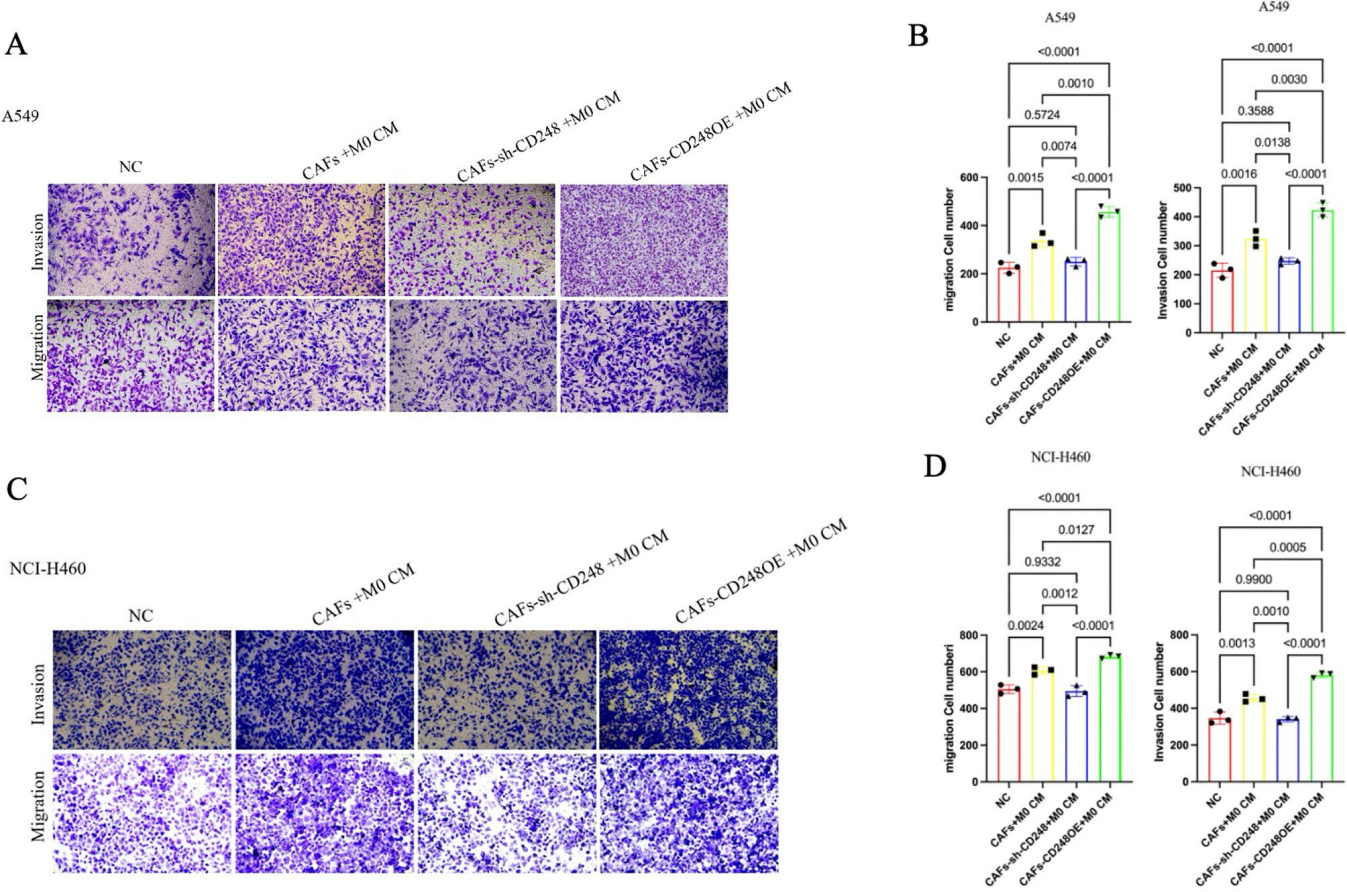
CD248-expressing CAFs induced M2-polarized macrophages to promote NSCLC migration and invasion. NSCLC cell migratory and invasive nature were characterized via Transwell assays. NSCLC cell lines A549 and NCI-H460 were co-cultured with conditional media of CAFs-sh-CON, CAFs-sh-CD248, CAFs-CD248OE treated THP-1 CM. (**A**) Transwell assay results examining the migration and invasion of A549 cells. (**B**) Quantification of the number of migration and invasion cells. (**C**) Transwell assay results examining the migration and invasion of NCI-H460 cells. (**D**) Quantification of the number of migration and invasion cells. We conducted three separate experiments. Data provided as mean ± SD.

**Figure 4:**
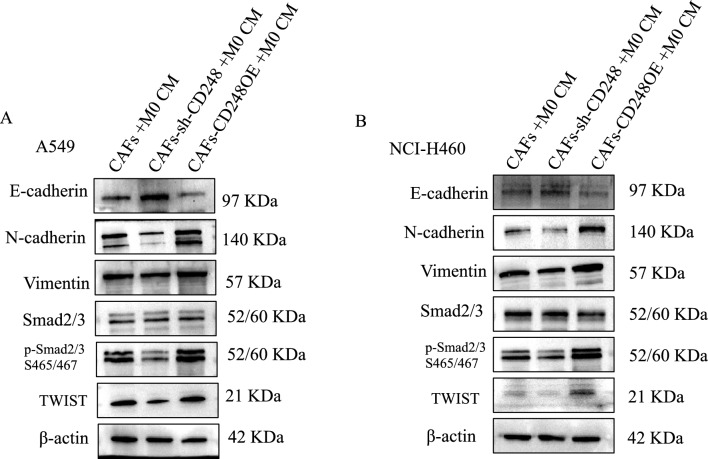
CD248-expressing CAFs induced M2-polarized macrophages to induce NSCLC cells EMT. (**A**) Western blot analysis illustrating the expression of E-cadherin, N-cadherin, Vimentin, Smad2/3, p-Smad2/3 and TWIST in A549 treated with conditional media of CAFs-sh-CON, CAFs-sh-CD248, CAFs-CD248OE treated THP-1 CM. Original blots are presented in Supplementary Fig. 2. (**B**) Western blot analysis illustrating the expression of E-cadherin, N-cadherin, Vimentin, Smad2/3, p-Smad2/3 and TWIST in NCI-H460 treated with conditional media of CAFs-sh-CON, CAFs-sh-CD248, CAFs-CD248OE treated THP-1 CM. To conserve both antibodies and membrane materials, the blots were cut prior to hybridization with antibodies. Original images of blots are presented in Supplementary Figs. 2, 3.

To examine whether the CD248-harboring CAFs activated M2-polarized macrophages to accelerate NSCLC invasive and migratory activities, the CAFs-sh-CON or CAFs-sh-CD248 CM and THP-1 cells were co-exposed to A549 and NCI-H460 cells. Using Transwell assessments, we verified that CD248-harboring CAFs increased A549 and NCI-H460 cellular invasion, relative to the CD248 deficient CM (*p* < 0.01) as shown in Fig. 3A–D. To elucidate the CD248-harboring CAFs activated M2-polarized macrophages-mediated facilitation of NSCLC invasion and migration via inducing NSCLC EMT, the NSCLC cell lines A549 and NCI-H460 were co-cultured with the CM derived from CAFs-sh-CON, CAFs-sh-CD248, or CAFs-CD248OE treated THP-1 cells, then we examined changes of EMT phenotype by measuring the protein expression of E-cadherin, N-cadherin, vimentin, Smad2/3, p-Smad2/3 and EMT-related transcription factors TWIST. The results revealed that A549 and NCI-H460 co-cultured with CM derived from CAFs-sh-CON or CAFs-CD248OE treated THP-1 cells had decreased the expression of E-cadherin,while the expression of N-cadherin, vimentin, p-Smad2/3 and TWIST were significantly increased (Fig. 4A,B). Based on these evidences, the CD248-harboring CAFs activated M2-polarized macrophages facilitated NSCLC invasion and migration via inducing EMT program of NSCLC cells.

### Fibroblast-specific *CD248* depletion promoted LC metastasis in vivo

**Figure 5:**
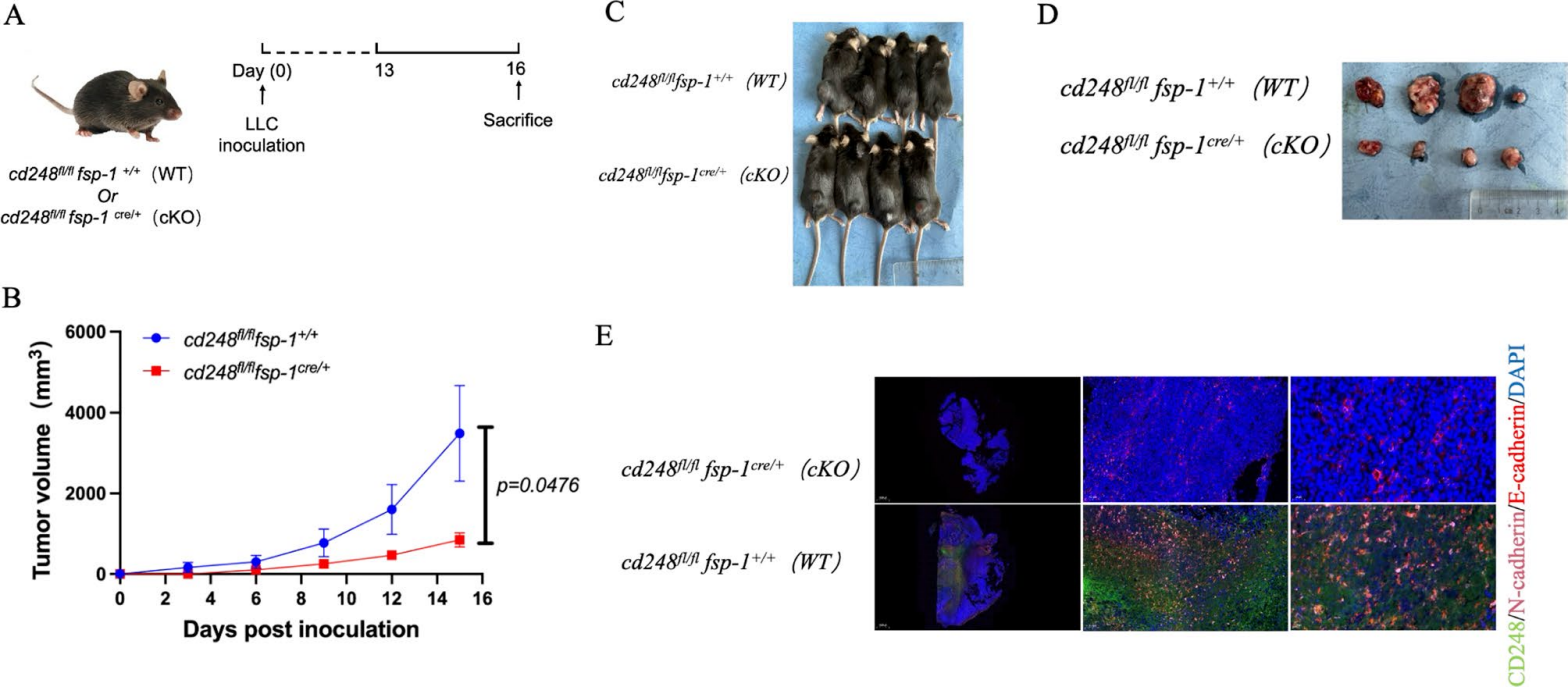
Fibroblasts-specific CD248 depletion inhibited Lung cancer (LC) EMT process in vivo. 5 × 10^5^ LLC cells injected into *cd248*^*fl/fl*^*fsp-1*^+*/*+^ (WT) and *cd248*^*fl/fl*^*fsp-1*^*cre/*+^ (cKO) mice to establish a LC metastasis model (n = 4 per group). (**A**) An illustration of the murine model. (**B**) Fluorescence intensity assessment per cohort. Data provided as mean ± SEM. *p* < 0.0001. (**C**,**D**) Images of the xenografts after inoculation with the LLC in WT and cKO mice. (**E**) Dual-IF staining results depicting the CD248 (green), N-cadherin (pink) and E-cadherin (red) in tumor tissue of WT and cKO mice. Scale bar, left: 100 μm; right: 20 μm.

LLC cells were administered to the subcutaneous of *cd248*^*fl/fl*^*fsp-1*^+*/*+^ (WT) and *cd248*^*fl/fl*^*fsp-1*^*cre/*+^ (cKO) mice (Fig. 5A). We meticulously monitored the tumor growth with tumor volume. Based on our findings, tumor growth was substantially enhanced in WT mice relative to CD248-deficient mice (*p* = 0.0476) (Fig. 5B–D). At experiment termination, mice were euthanized, and tumor were harvested from euthanized mice. Paraffin-embedded tissues from euthanized mice were stained with immunofluorescence (IF) for E-cadherin and N-cadherin. In WT murine tumor tissue, N-cadherin were highly expressed; a relatively faint fluorescent of E-cadherin signal was detected in tumor tissue (Fig. 5E). These results suggest that CD248-expressing-fibroblasts promoted EMT of LC.

## Discussion

EMT critically modulates metastasis, and it is strongly associated with worse cancer patient outcome. Increasing evidence indicates a complex association between the cells of the tumor and the tumor microenvironment, acting together to regulate tumor metastasis^18–20^. CAFs is a major TME constituent, and it utilizes numerous signaling networks to sustain tumor progression. However, its role in NSCLC cell EMT remains largely unelucidated.

However, owing to the diversity of fibroblasts, CAFs exhibit augmented phenotypic heterogeneity and they possess distinct phenotypes with distinct functions. In our previous studies, we discovered that CD248 is a potential marker of CAFs derived from NSCLC, CD248^+^CAFs secreting CXCL12 mediated NSCLC progression^14^. In this study, we also demonstrated that CD248^+^CAFs can mediate macrophages polarized to M2 type macrophages. M2-polarized macrophages secrete TGF-β. We found that CD248^+^CAFs mediated M2-polarized macrophages augments mesenchymal biomarker expressions, namely N-cadherin, and simultaneously suppresses epithelial biomarkers E-cadherin in NSCLC cells. In fibroblasts specific CD248 gene knock out mice lung cancer model, we demonstrated that CD248 knock out mice diminish the infiltration of M2-polarized macrophages and then inhibit NSCLC cells EMT process.

The two distinct macrophages express unique biomarkers and regulate discreet metabolic and genetic profile distribution. M1 macrophages synthesize and release proinflammatory cytokines, namely, IL-12, TNF-α, and IFN-γ, whereas, M2-polarized macrophages release the anti-inflammatory cytokines IL-10, IL-13 and IL-4^21–23^. M2-polarized macrophages activity is intricately linked to tumor proliferation and migration^24^. Several studies reported on the underlying pathways involved in the M2 macrophage-mediated regulation of tumor invasion and migration^25^. Wei et al. demonstrated that M2-polarized macrophages modulated the JAK2/STAT3/miR-506-33p/FoxQ1 network, increased CCL2 synthesis, and enhanced macrophage recruitment to activate EMT and accelerate cancer cell migration and invasion^26^. We found that CD248^+^CAFs induced macrophages polarized to M2 type macrophages, and then M2-polarized macrophages secreting TGF-β promotes NSCLC cells EMT process. But, herein, we have not yet examined the mechanism of M2-polarized macrophages secreting TGF-β induced NSCLC cells EMT process.

Our study demonstrated that CD248 is ubiquitous in NSCLC-originating CAFs. CD248^+^CAFs mediated macrophages polarized to M2 type macrophages. CD248^+^CAFs activated M2-polarized macrophages to enhance NSCLC cells EMT both in cellular and animal models. Our study emphasizes the importance and possible signaling networks of CD248^+^CAF in NSCLC progression and offers a novel strategy for improving the survival time of NSCLC patients.

## Supplementary Information

Supplementary Figures.

Supplementary Tables.

## Data Availability

The datasets used in the current study are available from the corresponding author upon reasonable request.

## Abbreviations

CAFs: Cancer-associated fibroblasts
EMT: Epithelial–mesenchymal transition
NAT: Normal adjacent tissues
NC: Negative control
NFs: Normal fibroblasts
NSCLC: Non-small cell lung cancer
STR: Short tandem repeat
TME: Tumour microenvironment
