# Author Correction: CD248-expressing cancer-associated fibroblasts induce epithelial–mesenchymal transition of non-small cell lung cancer via inducing M2-polarized macrophages

**DOI:** 10.1038/s41598-025-09189-3

**Published:** 2025-07-18

**Authors:** Jing Xiao, Zeyang Yang, Siyu Wang, Xinlei Liu, Yun Wang, Zuquan Hu, Zhu Zeng, Jieheng Wu

**Affiliations:** 1 Department of Immunology, Guizhou Medical University, Siya Road, Guiyang, 561113 China; 2 College of Stomatology, Guizhou Medical University, Guiyang, 561113 China; 3 Guizhou Prenatal Diagnosis Center, The Affiliated Hospital of Guizhou Medical University, Guiyang, 550001 China; 4 Immune Cells and Antibody Engineering Research Center of Guizhou Province, Key Laboratory of Biology and Medical Engineering, Guizhou Medical University, Guiyang, 561113 China; 5 Key Laboratory of Infectious Immune and Antibody Engineering of Guizhou Province, Engineering Research Center of Cellular Immunotherapy of Guizhou Province, School of Biology and Engineering/School of Basic Medical Sciences, Guizhou Medical University, Guiyang, 561113 China; 6 The State Key Laboratory of Cancer Biology, Department of Biochemistry and Molecular Biology, The Fourth Military Medical University, Xi’an, 710032 China; 7 Tumor Immunotherapy Technology Engineering Research Center of Guizhou Medical University, Guizhou Medical University, Guiyang, 561113 China

Correction to: *Scientific Reports* 10.1038/s41598-024-65435-0, published online 21 June 2024

The original version of the Article contained errors.

As a result of an error introduced during the final figure formatting stage, the Vimentin western blot data panels were mistakenly duplicated across replicates for both NCI-H460 and A549 during figure assembly.

The NCI-H460 Vimentin condition in Figure 4B was mistakenly a duplication of the A549 Vimentin condition in Figure  4A. In the Supplementary Figures file, the NCI-H460 Vimentin condition in Supplementary Figure 3 was mistakenly a duplication of the A549 Vimentin condition in Supplementary Figure 2.

**Fig. 4:**
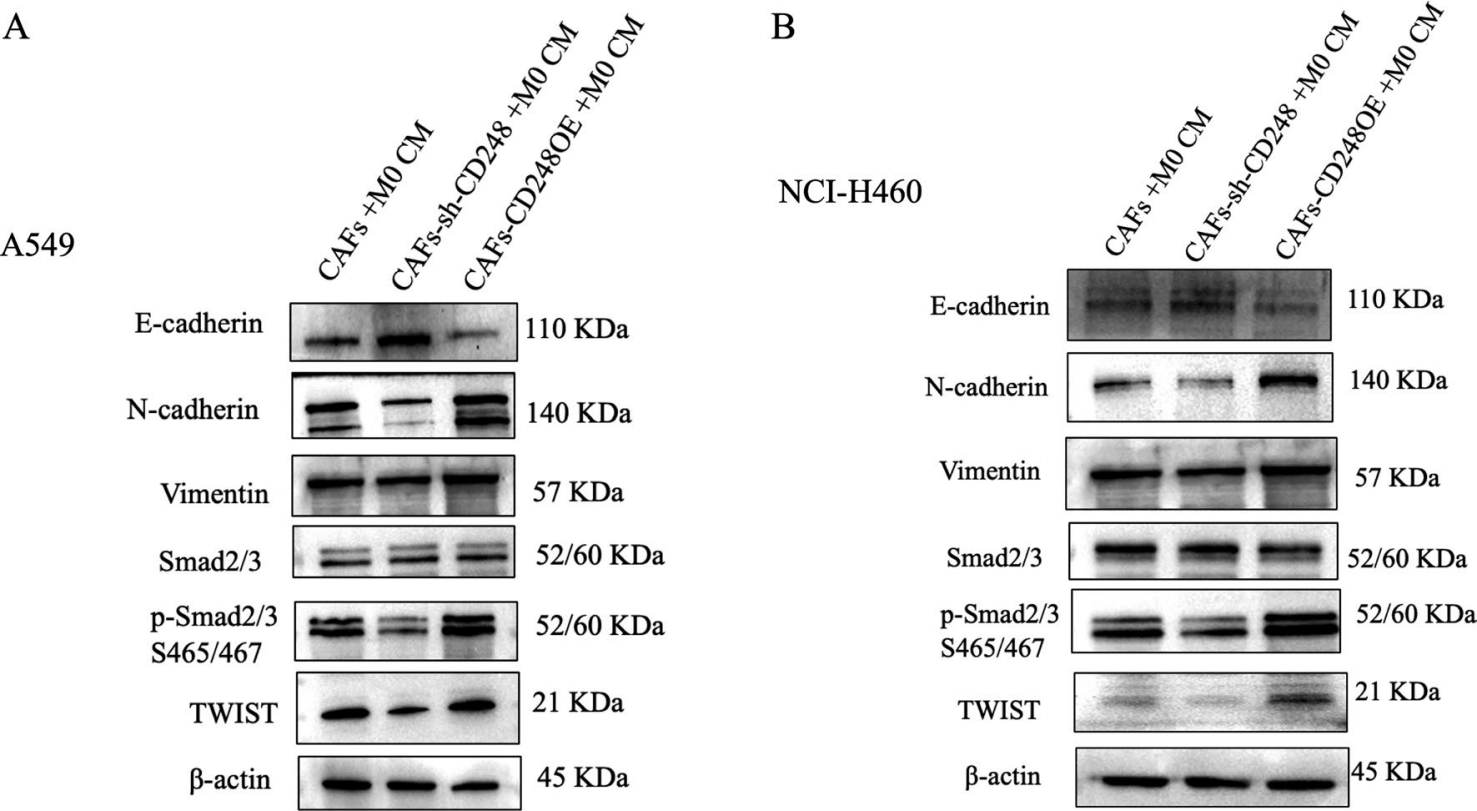
CD248-expressing CAFs induced M2-polarized macrophages to induce NSCLC cells EMT. (**A**) Western blot analysis illustrating the expression of E-cadherin, N-cadherin, Vimentin, Smad2/3, p-Smad2/3 and TWIST in A549 treated with conditional media of CAFs-sh-CON, CAFs-sh-CD248, CAFs-CD248OE treated THP-1 CM. Original blots are presented in Supplementary Fig. 2. (**B**) Western blot analysis illustrating the expression of E-cadherin, N-cadherin, Vimentin, Smad2/3, p-Smad2/3 and TWIST in NCI-H460 treated with conditional media of CAFs-sh-CON, CAFs-sh-CD248, CAFs-CD248OE treated THP-1 CM. To conserve both antibodies and membrane materials, the blots were cut prior to hybridization with antibodies. Original images of blots are presented in Supplementary Figs. 2, 3.

The original Figure 4 and accompanying legend appear below.

The original Article has been corrected.

## Supplementary Information

Supplementary Figures.

