# Supplementary material for: CD248-expressing cancer-associated fibroblasts induce epithelial–mesenchymal transition of non-small cell lung cancer via inducing M2-polarized macrophages
Source: Sci Rep. 2024 Jun 21;14:14343. doi: 10.1038/s41598-024-65435-0 (PMC11192924; doi:10.1038/s41598-024-65435-0)
Supplement: Supplementary file 2 — Supplementary Tables. [file 41598_2024_65435_MOESM2_ESM.docx]

**Supplementary Table**

**Primer sequence for q-PCR**

| gene name | Forward primer: | Reverse primer: |
| --- | --- | --- |
| human FAP | 5’- TCTAAGGAAAGAAAGGTGCCAA -3’ | 5’- GATCAGTGCGTCCATCATGAAG -3’ |
| human Vimentin | 5’-GACAATGCGTCTCTGGCACGTCTT-3’ | 5’- TCCTCCGCCTCCTGCAGGTTCTT-3’ |
| human CD206 | 5’-GGACGTGGCTGTGGATAAAT-3’ | 5’-ACCCAGAAGACGCATGTAAAG-3’ |
| human CD248 | 5’-ACTACGTTGGTGGCTTCGAG-3’ | 5’-CACTGAGGAGTGGTAGGGGA-3’ |
| human GAPDH | 5’-GGAGCGAGATCCCTCCAAAAT-3’ | 5’-GGCTGTTGTCATACTTCTCATGG-3’ |
