# Supplementary material for: Author Correction: CD248-expressing cancer-associated fibroblasts induce epithelial–mesenchymal transition of non-small cell lung cancer via inducing M2-polarized macrophages
Source: Sci Rep. 2025 Jul 18;15:26097. doi: 10.1038/s41598-025-09189-3 (PMC12274494; doi:10.1038/s41598-025-09189-3)
Supplement: Supplementary file 1 — Supplementary Figures. [file 41598_2025_9189_MOESM1_ESM.doc]

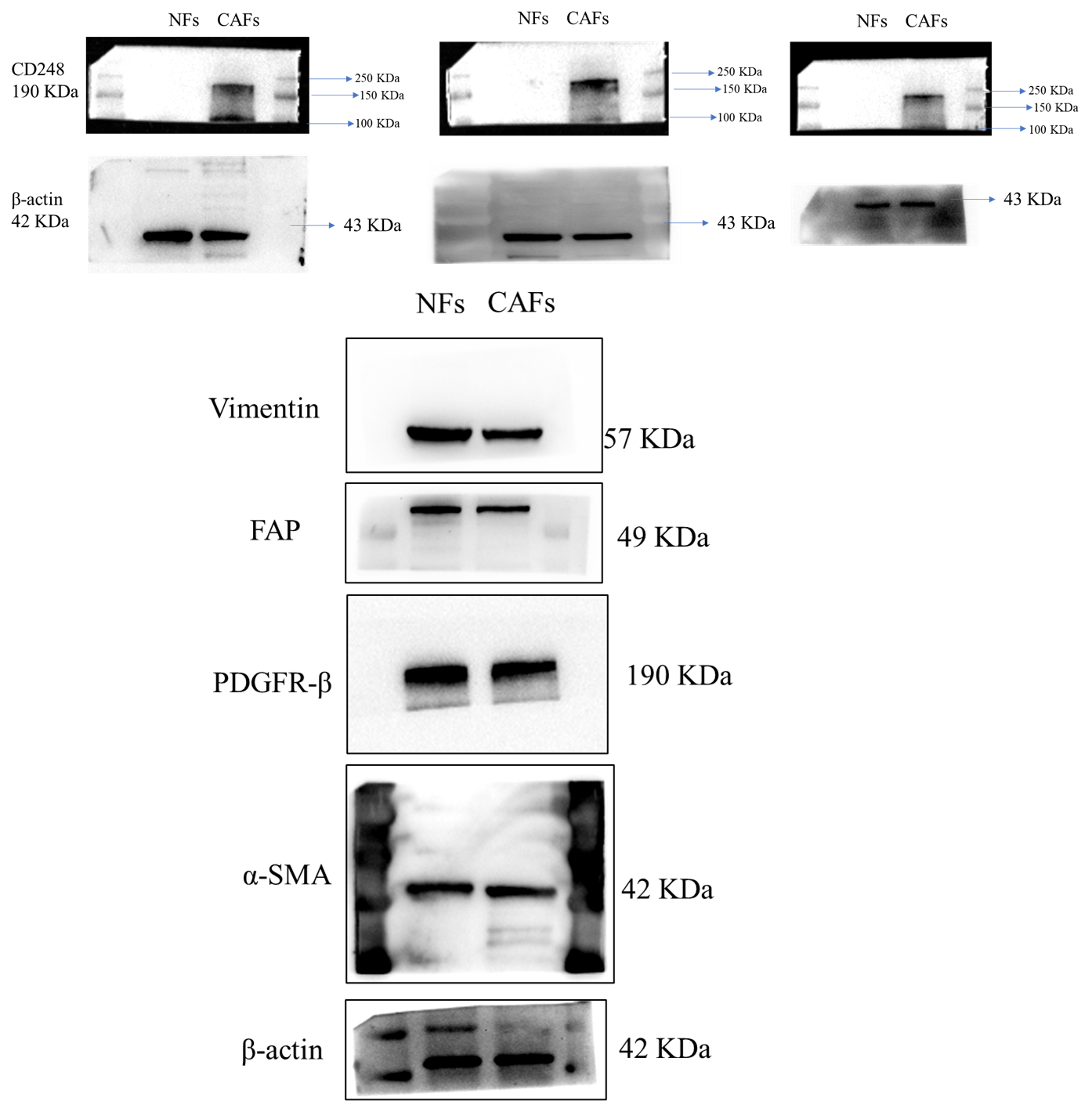


Supplementary FigureS1. The exression of CD248, PDGFR-β, α-SMA,Vimentin, FAP and β-actin was tested by western blotting in NFs and CAFs. β-actin was used as internal control. These cropped blots are used in the main figure (Figure 1) and these full-length blots are included in the supplementary figure.


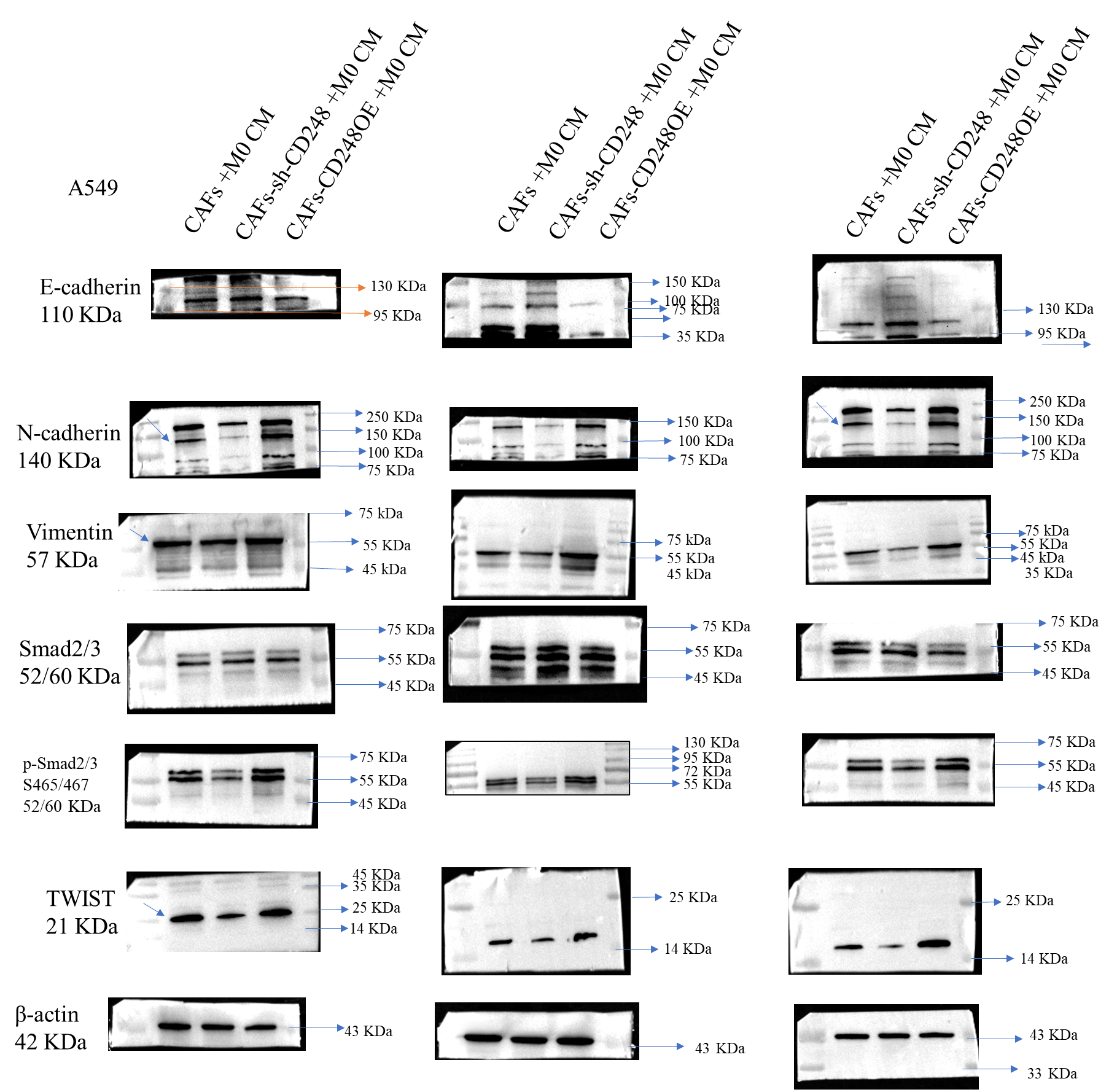
Supplementary FigureS2. The exression of E-cadherin, N-cadherin, Vimentin, Smad2/3, p-Smad2/3 and TWIST was tested by western blotting in A549 tumor cells. β-actin was used as internal control. These cropped blots are used in the main figure (Figure 4 A) and these full-length blots are included in the supplementary figure.


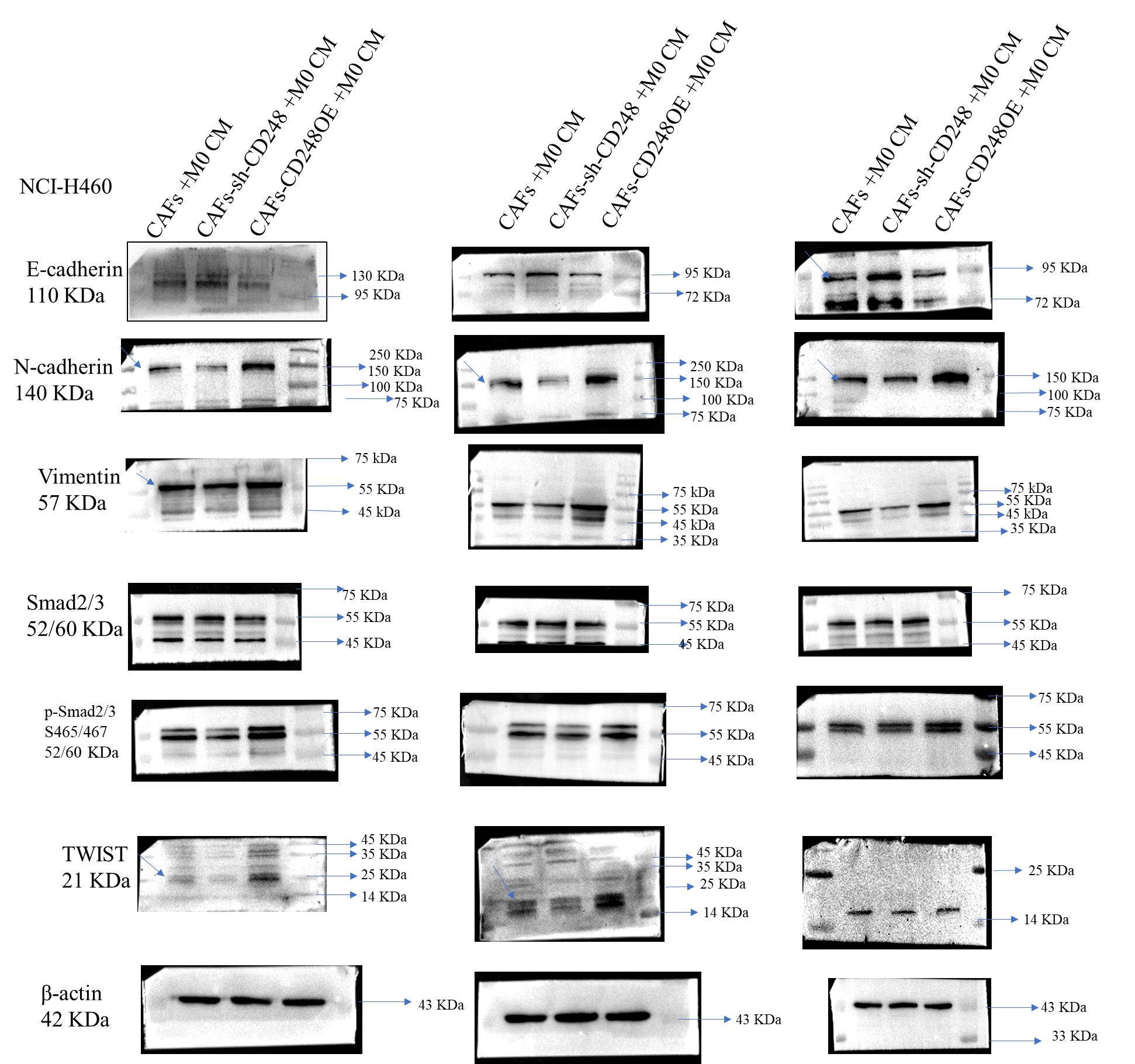


Supplementary FigureS3. The exression of E-cadherin, N-cadherin, Vimentin, Smad2/3, p-Smad2/3 and TWIST was tested by western blotting in NCI-H460 tumor cells. β-actin was used as internal control. These cropped blots are used in the main figure (Figure 4 B) and these full-length blots are included in the supplementary figure.
